# In Situ Growth of Ca^2+^-Based Metal–Organic Framework on CaSiO_3_/ABS/TPU 3D Skeleton for Methylene Blue Removal

**DOI:** 10.3390/ma13194403

**Published:** 2020-10-04

**Authors:** Zhen Liu, Xinshu Xia, Wei Li, Liren Xiao, Xiaoli Sun, Fubin Luo, Qinghua Chen, Qingrong Qian

**Affiliations:** 1 College of Chemistry and Materials, Fujian Normal University, Fuzhou 350007, China; liuzhen3681@126.com; 2 Engineering Research Center of Polymer Green Recycling of Ministry of Education, Fujian Normal University, Fuzhou 350007, China; sunxiaoli@fjnu.edu.cn (X.S.); luofubin@fjnu.edu.cn (F.L.); cqhuar@fjnu.edu.cn (Q.C.); 3 College of Environmental Science and Engineering, Fujian Normal University, Fuzhou 350007, China; LW13860055277@163.com; 4 Fujian Key Laboratory of Pollution Control & Resource Reuse, Fujian Normal University, Fuzhou 350007, China

**Keywords:** 3D printing, metal–organic framework, in-situ growth, adsorption, methylene blue

## Abstract

The work reports a novel strategy for combining polymers and metal–organic frameworks (MOFs) into composites for adsorption applications. Calcium silicate (CaSiO_3_) was introduced into acrylonitrile butadiene styrene/thermoplastic polyurethane (ABS/TPU) alloy, and the CaSiO_3_/ABS/TPU skeleton was fabricated by 3D printing technology. The Ca-MOF was directly loaded on the surface of acetone-etched 3D skeleton by in-situ growth method. The obtained 3D skeleton was characterized and the performance of methylene blue (MB) adsorption was determined. It is clear that Ca-MOF is successfully loaded on the surface of 3D skeleton due to the presence of CaSiO_3_. The MB adsorption ratios of the solutions with initial concentrations of 50, 100 and 200 mg/L at the equilibrium time (5 h) are 88%, 88% and 80%, respectively, revealing good MB adsorption performance of the 3D skeleton. The MB adsorption ratio remains 70% at six runs of adsorption–desorption experiment, indicating the excellent recovering property of the skeleton. The results show that the prepared CaSiO_3_/ABS/TPU 3D skeleton is a candidate adsorbent for printing and dyeing effluent treatment.

## 1. Introduction

In recent years, the design and synthesis of metal–organic frameworks (MOFs) have undergone tremendous development [1,2,3,4,5,6] due to their potential applications in gas storage [3], molecular separation [7], heterogeneous catalysis [4], drug delivery [8], and functional devices [9,10]. Besides, MOFs are also applied to the water purification process [11] because of their large specific surface area and exposed metal sites. However, the crystallinity of MOFs causes its form of powders, which greatly limits their practical application. Therefore, consolidating MOFs into a monolithic material and promoting their recycling becomes a critical topic [12,13].

As a kind of additive manufacturing technology, 3D printing has drawn great attention in various fields, attributed to its advantages in manufacturing complex shapes and high-precision models, maximum material saving, flexible design and individual customization. It is worth noting that the 3D printing technique has been used in extruding composite materials into customizable shapes for adsorption separation [14], biomedicine [15], catalyst [16], and other fields [17]. It is also a promising way to transform MOFs from powder into device and applies to slow drug release [3], lithium batteries [18], gas separation [19,20,21,22,23] and wastewater treatment [12,24,25,26]. Halevi et al. [27] used thermoplastic materials as host materials to fabricate a 3D, flexible, and hydrolytically stable MOF-embedded thermoplastic frame. Michael Bible [19] directly incorporated HKUST−1 and ZIF−8 into 3D printed filaments, and then printed the model containing MOFs through a printer. Rui Pei [12] fabricated Cu-BTC/biocompatible polymer scaffolds with 3D printing technique, which could be utilized as excellent adsorbents towards organic dyes. Unfortunately, the approach leads to most of MOFs being embedded in the polymer and inhibiting the MOFs playing their roles. Thus, growing MOFs directly on the surface of the 3D skeleton seems to be an alternative approach. Wang [26] successively immersed the acrylonitrile butadiene styrene (ABS) skeleton in organic solution and metal ions to prepare Cu-BTC/ABS 3D skeleton. The skeleton showed high capacity for removal of MB from aqueous solution. Shi [24] successfully grow Cu-BTC in situ on PLA membranes and applied it to the treatment of dyes in reclaimed wastewater. However, most of the reported 3D printing MOF materials are derived from Co, Zn, Cu and possess disadvantages due to the long reaction, complex operation and the secondary pollution caused by the metal ion solution. Recently, calcium salts are selected as a metal formation precursor for MOFs because of their non-toxic, low-cost and high bio-melting properties [28,29]. Kenji et al. [30] have successfully synthesized a new Ca-MOF with calcium carbonate and 2,5-Dihydroxy−1,4-benzoquinone. They used a marine organism shell-derived calcium carbonate as a source of metal and converted it into a MOFs system by coordination replication.

In this study, CaSiO_3_ was introduced in ABS/thermoplastic polyurethane (TPU) melted blend in a twin-screw extruder, and CaSiO_3_/ABS/TPU filament was extruded on a mini filament system for 3D printing. MOFs were loaded directly on the surface of acetone-etched ABS/TPU/CaSiO_3_ 3D printed skeleton via an in-situ growth method. The obtained Ca-MOF/ABS/TPU 3D skeleton indicates an alternative absorbent for the treatment of the printing and dyeing wastewater.

## 2. Materials and Methods 

### 2.1. Materials

The acrylonitrile-butadiene-styrene copolymer (ABS) AG10AP was purchased from Nature Taiwan Chemical Fiber Co., Ltd. (Taiwan, China). The thermoplastic polyurethane (TPU) 5377A was obtained from Bayer, Germany. Microporous calcium silicate (CaSiO_3_, 1000 mesh) was provided by Shanxi jade Zhuxin Materials Technology Co., Ltd. (Shanxi, China). Both ABS and TPU pellets were dried under vacuum at 80 °C for 12 h to remove moisture before use, and CaSiO_3_ was dried under vacuum at 120 °C for 12 h prior to the melt blending. The 2,5-Dihydroxy−1,4-benzoquinone (H_2_dhbq) was obtained from Aladdin Chemical Reagent Co., Ltd. (Shanghai, China). Acetone, methanol, ethanol, methylene blue (MB, analytical grade), sodium chloride (NaCl), hydrochloric acid (HCl), and sodium hydroxide (NaOH) were purchased from Fuchen Chemical Reagent Co., Ltd. (Tianjin, China), and used as received.

### 2.2. Immobilization of CaSiO_3_ on the ABS/TPU 3D Filaments

**Figure 1:**
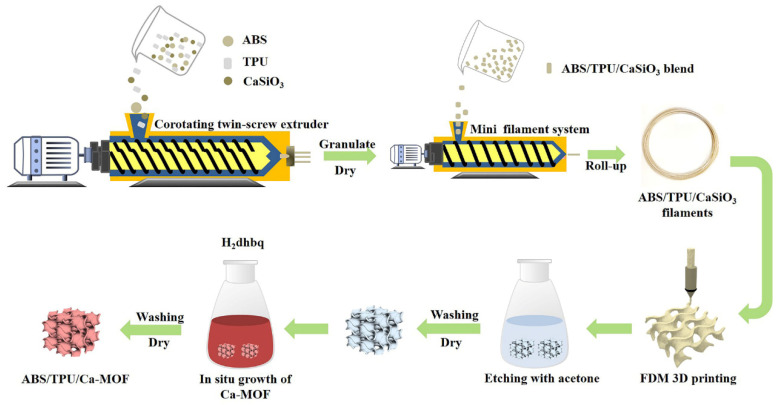
Schematic diagram of Ca-MOF/ABS/TPU 3D skeleton.

As shown in Figure 1, the dried ABS, TPU and CaSiO_3_ were premixed on a homogenizer(Nissin Electrical Co., Ltd., Jiangsu, China) and then extruded on a POTOP MEDI−22/40 co-rotating twin-screw extruder with a screw diameter of 22 mm (POTOP Experimental Analysis Instrument Co., Ltd., Guangzhou, China). The temperatures of the extruder were set to be 100, 125, 145, 160, 170, 175, 175, 170, 155 and 145 °C from the feed zone to the die, respectively. The rotation speed of the screw was 200 rpm. The strap of blends was cooled, granulated and dried in a vacuum oven(Yongguangming Medical Instrument Co., Ltd., Beijing, China) at 60 °C overnight. The mass rate of ABS/TPU was fixed at 80/20 and the dosage of CaSiO_3_ powder was 40 phr. The dried CaSiO_3_/ABS/TPU pellets were employed as raw materials, and filaments were extruded on a two-heating zone mini filament system (Wellzoon Type C, Mistar Technology Co., Ltd., Shenzhen, China) with a screw speed of 15 rpm at temperatures of 155 and 175 °C. The obtained CaSiO_3_/ABS/TPU filaments possess an average diameter of 1.75 mm.

### 2.3. Preparation of CaSiO_3_/ABS/TPU 3D Skeleton by FDM Printing Process

Two scaffold models, square and the spiral skeleton were designed using a 3D CAD program. And the scaffolds were printed using the obtained CaSiO_3_/ABS/TPU filament as a feeder on an FDM printer (MD2030, Sengong Technology Co., Ltd., Shenzhen, China), respectively. The nozzle temperature was 200 °C, and the platform temperature was 60 °C. The printing speed was 50 mm/s, the layer thickness was 0.2 mm, and the nozzle diameter was 0.40 mm. The scaffolds were acetone-etched prior to in situ growth of MOFs.

### 2.4. Preparation of Ca-MOF/ABS/TPU 3D Skeleton

**Figure 2:**
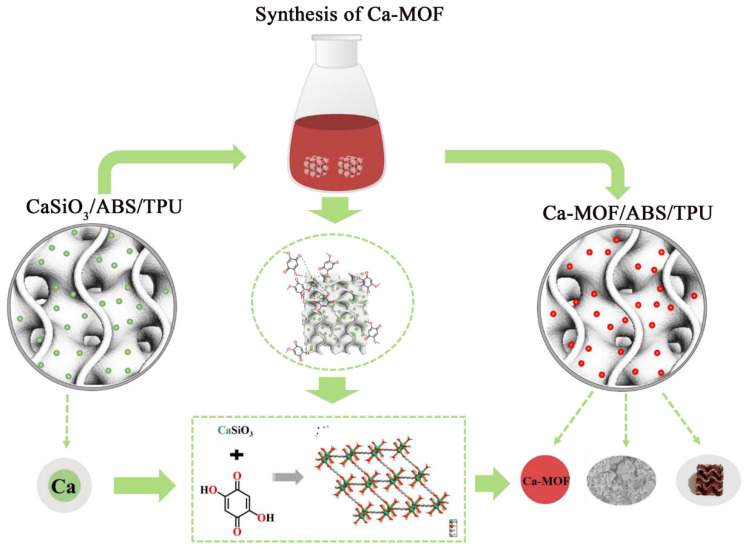
Schematic diagram for the formation of Ca-MOF on the surface of ABS/TPU 3D skeleton.

The prepared CaSiO_3_/ABS/TPU 3D printed skeleton was etched in acetone for 5 min to expose more CaSiO_3_ on the surface for providing MOFs loading substrate and growth sites. The clean and dried acetone-etched skeleton was immersed in a solution of H_2_dhbq (0.1 g/L, 50 mL) for 12 h to grow Ca-MOF on the surface of the skeleton (Figure 2). The Ca-MOF/ABS/TPU 3D skeleton then was washed with methanol and dried at 80 °C overnight. The ABS/TPU 3D skeleton without MOF denoted as ABS/TPU−2 3D skeleton was also prepared for comparison. Pure Ca-MOF powder without ABS/TPU was synthesized according to the method in the literature [30].

$$CaSiO_{3}+H_{2}dhbq\to \left[Cadhbq{\left(H_{2}O\right)}_{2}\cdot H_{2}O\right]n+SiO_{2}+H_{2}O$$

### 2.5. Adsorption of Methylene Blue

The adsorption of MB on the Ca-MOF/ABS/TPU 3D skeleton in aqueous solution was performed in a batch experiment. Typically, 0.9 g Ca-MOF/ABS/TPU was added to 50 mL MB aqueous solution with various initial concentrations (50, 100 or 200 mg/L). The mixture was shaken at room temperature for 300 min and the UV–vis spectra of MB were recorded at different interval times. The MB concentration was quantified based on the UV–vis absorption intensity at 664 nm. The adsorption of MB (100 mg/L) on the ABS/TPU−2 was also carried out for comparison. The square and spiral skeleton models were employed to analyze the experimental data of MB adsorption. Then the MB sorption was investigated in solutions of various pH values (pH = 3–10). The adsorption thermodynamics experiment was investigated at varying temperatures of 5, 25, 45 °C. Then the adsorption kinetics experiment was performed. The removal efficiency and adsorption quantity of the MB were calculated by applying Equations (1) and (2), respectively:

(1) $$R(\%)=\frac{\left(C_{0}-C_{e}\right)}{C_{0}}\times 100\%$$

(2) $$q_{e}=\frac{\left(C_{0}-C_{e}\right)}{m}\times V$$

where *R* and *q_e_* are the removal efficiency and amount of dye taken up by the Ca-MOF/ABS/TPU 3D skeleton, and *C*_0_ and *C_e_* are concentration of the dye at the initial and equilibrium, respectively. *V* is the volume of the MB solution (L), and m is the weight of the Ca-MOF/ABS/TPU 3D skeleton.

### 2.6. Reusability

The reusability of Ca-MOF/ABS/TPU 3D skeleton was evaluated via solvent desorption techniques. Methanol was used as an eluent to regenerate Ca-MOF/ABS/TPU. The Ca-MOF/ABS/TPU sample adsorbed with MB was immersed in methanol with an ultrasonic vibration. It was then stopped to replace fresh methanol until the solution showed colorless. Then, the MB adsorption behavior of the regenerated Ca-MOF/ABS/TPU was determined again. The experiment was carried out for six runs.

### 2.7. Characterization

The morphology of 3D skeletons before and after growth of MOFs on the surface was obtained on a field emission scanning electron microscope (SEM) (JSM−7500F; JEOL, Tendo city, Yamagata, Japan) with the acceleration voltage of 10 kV. The sample was placed on a carbon sheet and sputter coated with gold for 120 s. The energy dispersive spectrometer (EDS) (JSM−7500F; JEOL, Tendo city, Yamagata, Japan) of the samples was also recorded on it. 

The Fourier Transform infrared spectroscopy (FTIR) spectra of Ca-MOF powder, CaSiO_3_/ABS/TPU and Ca-MOF/ABS/TPU 3D skeletons were recorded in the range of 4000–400 cm^−1^ on a FTIR spectrometer (Nicolet Is10, Thermo Scientific, Massachusetts, USA). The sample was grounded with KBr and pressed into a pellet prior to the determined. 

X-ray diffraction measurement was performed on an X-ray powder diffractometer (D8, Bruker, Germany) with Cu Ka radiation (Phaser λ = 0.179 nm). The scanning step size was 0.02 degrees/step and the scanning rate was 147.4 s/step. 

The thermal characteristics of CaSiO_3_/ABS/TPU and Ca-MOF/ABS/TPU composites were analyzed on a Thermogravimetric (TG) analyzer (Q50, TA Instruments, New Castle, PA, USA) under nitrogen atmosphere (50 mL/min). The temperature was raised from 30 to 800 °C with a heating rate of 10 °C min^−1^. 

The specific surface area of the samples was tested on a Brunauer-Emmet-Teller (BET) nalyzer (BELSORP-mini, MicrotracBEL, Osaka, Japan). The sample was degassed at the temperature of 80 °C for 3 h prior to the N_2_ adsorption test. 

The pH of the point of zero charge (pHpzc) for Ca-MOF/ABS/TPU 3D skeleton was measured by the pH drift method. Then, 50 mL of 0.1 mol L^−1^ NaCl was adjusted to the range of 3.0–14.0 using 1 mol/L HCl or 1 mol/L NaOH additions. The C\a-MOF/ABS/TPU 3D skeleton was added to solution stirred for 12 h and the final pH was recorded using a pH-meter (PHS−3C, Rex Electric Chemical, Shanghai, China). The pH_equilibrium_ was plotted against pH_initial_, the point at which the curve crossed the line pH_equilibrium_ = pH_initial_ was taken as the PZC (pHpzc).

## 3. Results and Discussion

### 3.1. Characterization of Ca-MOF/ABS/TPU 3D Skeleton

**Figure 3:**
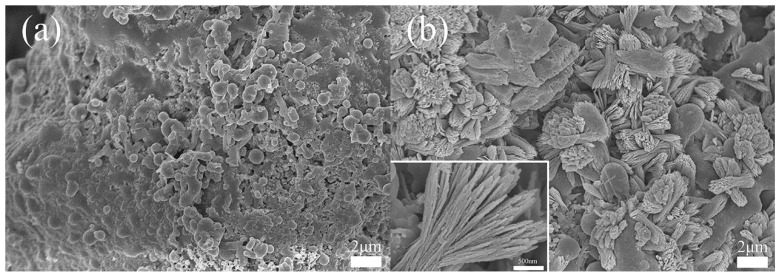
SEM images of (**a**) CaSiO_3_/ABS/TPU (**b**) Ca-MOF/ABS/TPU.

The morphology of CaSiO_3_/ABS/TPU and Ca-MOF/ABS/TPU 3D skeletons are shown in Figure 3.

In Figure 3a, the CaSiO_3_/ABS/TPU 3D skeleton gives a coarse surface due to the exposing of TPU spheres and CaSiO_3_ particles by removal of ABS via acetone etching. In Figure 3b, a large number of crystals are observed to grow on the surface of the 3D skeleton by soaking the CaSiO_3_/ABS/TPU 3D skeleton in the solution of H_2_dhbq. The presence of CaSiO_3_ on the surface provides sites for the crystals to grow. The high-resolution SEM image of Ca-MOF crystals (Figure 3b, insert) shows that the morphology of crystals is flower-like with a large specific surface area. The features are considered to be beneficial for dye adsorption.

**Figure 4:**
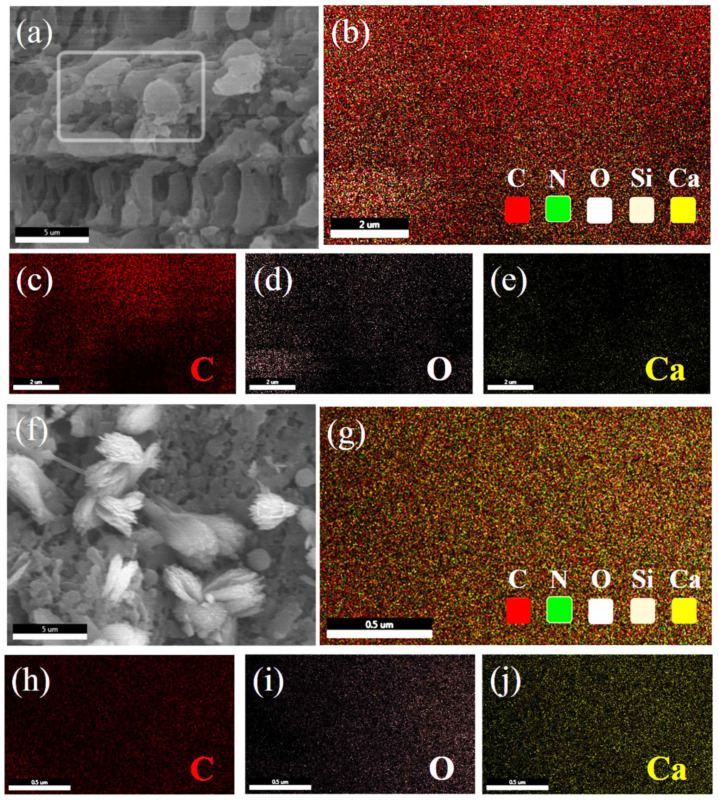
SEM images of (**a**) CaSiO_3_/ABS/TPU and (**f**) Ca-MOF/ABS/TPU, EDS element mappings of (**b**–**e**) CaSiO_3_/ABS/TPU and (**g**–**j**) the crystal on the Ca-MOF/ABS/TPU surface.

Figure 4 shows the surface scan patterns of CaSiO_3_/ABS/TPU and Ca-MOF/ABS/TPU 3D skeletons. 

For the CaSiO_3_/ABS/TPU 3D skeleton (Figure 4b–e), the contents of C, O and Ca are calculated to be 61, 23 and 6 wt.%, respectively. The high content of C is attributed to the high proportion of ABS and TPU in the 3D skeleton. For the crystal on the Ca-MOF/ABS/TPU 3D skeleton surface, the contents of C, O and Ca are found to be 28, 45 and 29 wt.%, suggesting that the crystal on the skeleton surface is Ca-MOF (CaC_6_H_8_O_7_), which will be confirmed by XRD analyses. The result reveals that Ca-MOF has been successfully loaded on the surface of the CaSiO_3_/ABS/TPU 3D skeleton via in-situ growth method.

**Figure 5:**
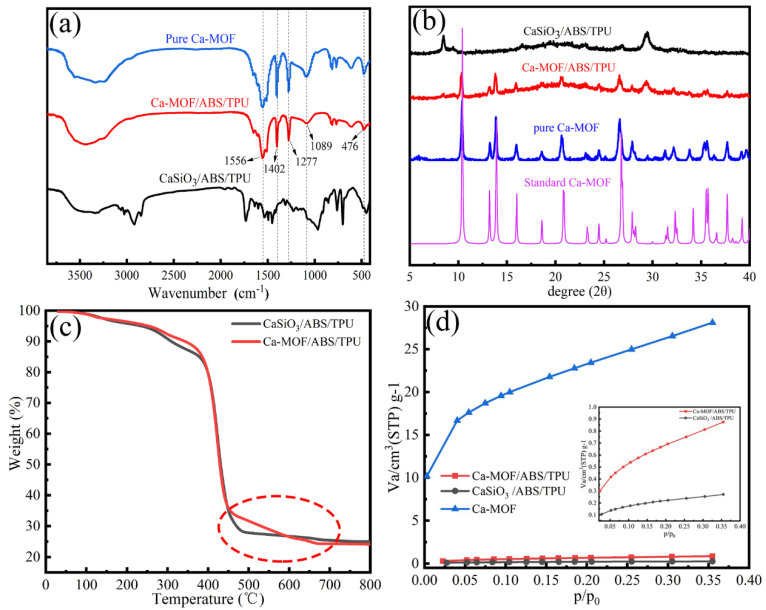
(**a**) FTIR spectra, (**b**) XRD patterns, and (**d**) N_2_ adsorption isotherms of CaSiO_3_/ABS/TPU, Ca-MOF/ABS/TPU and pure Ca-MOF, (**c**) TG curves of CaSiO_3_/ABS/TPU and Ca-MOF/ABS/TPU.

FTIR spectra, XRD patterns, TG curves and N_2_ adsorption isotherms are given in Figure 5.

In Figure 5a, the characteristic absorption peaks at 476, 1089, 1277, 1402 and 1556 cm^−1^ appear in the Ca-MOF/ABS/TPU 3D skeleton. The peaks at 476 and 1277 cm^−1^ are assigned to the bending vibration of benzoquinone and stretching vibration of C–O in the cyclic ketone. The peak at 1556 cm^−1^ is considered to be the stretching vibration of unsaturated ketone in the phenylhydrazine. The peak gives a red shift due to the conjugating of carbonyl group with olefinic bond [31]. The peak at 1089 cm^−1^ is considered to be the stretching vibration of the cyclic secondary alcohol in phenylhydrazine, while the peak at 1402 cm^−1^ is assigned to O–H bending vibration of the hydrated Ca^2+^ in MOF [31]. The results indicate that Ca-MOF has been successfully loaded on the surface of the 3D skeleton.

In Figure 5b, the XRD pattern of Ca-MOF is consistent with that reported in literature [30], suggesting the successful preparation of Ca-MOF. The pattern of Ca-MOF/ABS/TPU gives a similar shape to that of CaSiO_3_/ABS/TPU. Both of them give broad peaks at 2θ = 19.7°, which is due to the amorphous structure of the copolymer, and the peak at 2θ = 29.48° is attributed to the presence of CaSiO_3_ according to the standardized PDF 00–029−0331 and JCPDS−36–1451 [32]. However, some extra sharp peaks appeared at 2θ = 10.38°, 13.22°, 13.84°, 15.96°, 20.61°, 26.54°, 36.31° and 37.69° in the patter of Ca-MOF/ABS/TPU, which are assigned to pure Ca-MOF with a monoclinic crystal system (space group C2/m) [30]. 

Figure 5c displays the TG curves of the CaSiO_3_/ABS/TPU and Ca-MOF/ABS/TPU 3D skeletons. The two curves are similar in shape due to the same substrate material. A weak weight loss occurs in the temperature range of 100–400 °C, attributing to the ordinary dehydration and evaporation of interlayer water in mineral microporous CaSiO_3_. The violent weight loss began at 400 °C and is attributed to the structural decomposition of the polymer via forming butadiene, styrene and monomeric acrylonitrile [33]. Ca-MOF/ABS/TPU gives a significant weight loss but CaSiO_3_/ABS/TPU gives little weight loss in the temperature range of 450–650 °C, indicating the occurrence of Ca-MOF thermal decomposition in the temperature range [32]. The amount of Ca-MOF grown on the framework is calculated to be about 3.2 wt.%. The value is much higher than that of 0.15 wt.% reported in literature [26]. 

Figure 5d shows the N_2_ adsorption curves of Ca-MOF, CaSiO_3_/ABS/TPU and Ca-MOF/ABS/TPU under relative pressure between 0.05 and 0.35. The calculated BET specific surface areas of materials are 0.864 m^2^·g^−1^ for CaSiO_3_/ABS/TPU and 2.708 m^2^·g^−1^ for Ca-MOF/ABS/TPU, respectively, revealing that the introduction of Ca-MOF in the CaSiO_3_/ABS/TPU 3D skeleton leads to increasing surface area to some extent. It is worth noting that the specific surface area value of 2.708 m^2^·g^−1^ for Ca-MOF/ABS/TPU approaches the value calculated by multiplying the Ca-MOF dosage of 3.2 wt.% in the Ca-MOF/ABS/TPU 3D skeleton by its specific surface area of 83.42 m^2^·g^−1^. Compared to the MOFs reported in the literatures, the resultant Ca-MOF has a smaller specific surface area, and the lower amount loaded on the skeleton, resulting in a smaller the specific surface area of Ca-MOF/ABS/TPU.

### 3.2. Effects of Adsorption Conditions

**Figure 6:**
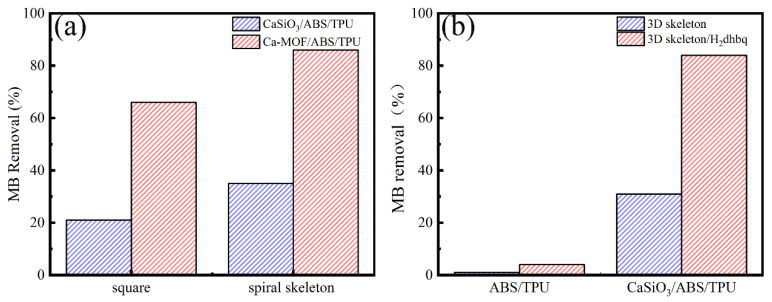
(**a**) Comparison of different models, (**b**) MB adsorption capacity of ABS/TPU and CaSiO_3_/ABS/TPU skeletons.

**Figure 7:**
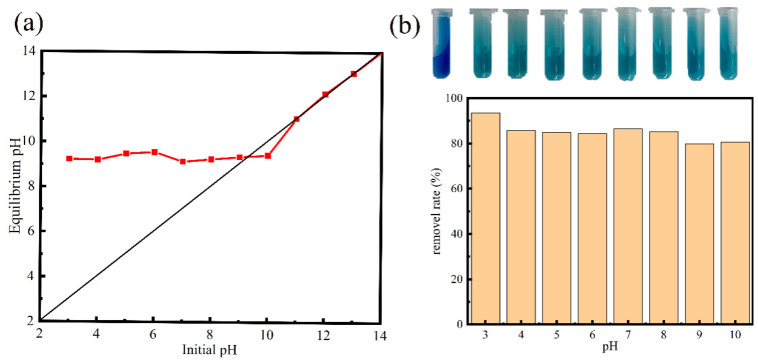
(**a**) pH drift method to determine the pH of the point of zero charge (pHpzc) for the Ca-MOF/ABS/TPU 3D skeleton. (**b**) Effect of pH on the MB adsorption by the Ca-MOF/ABS/TPU 3D skeleton (the initial MB concentration was 100 mg/L).

Figure 6 and Figure 7 show the adsorption behaviors of MB on CaSiO_3_/ABS/TPU and Ca-MOF/ABS/TPU skeletons.

Figure 6a shows the removal ratio of MB on spiral skeleton and square of various composites. It is clear that the removal ratios of MB on the CaSiO_3_/ABS/TPU spiral skeleton and square one are 35% and 21%, whereas those on the Ca-MOF/ABS/TPU spiral skeleton and square one are 86% and 66%. Ca-MOF/ABS/TPU shows higher MB removal ability than CaSiO_3_/ABS/TPU due to the introduction of Ca-MOF. In addition, the spiral skeleton gives higher MB removal ability than the square attributed to the increment of specific surface area by structure design.

Figure 6b shows the adsorption of MB on ABS/TPU, ABS/TPU−2, CaSiO_3_/ABS/TPU, and Ca-MOF/ABS/TPU 3D skeletons. The removal ratio of MB by ABS/TPU and ABS/TPU−2 was 1% and 4%, showing no significant difference. Little MOF loads on the surface of ABS/TPU 3D skeleton due to the lack of active sites. However, the removal ratio of MB by CaSiO_3_/ABS/TPU and Ca-MOF/ABS/TPU skeletons increases from 31% to 84%. The formation of Ca-MOF on the skeleton surface results in an obvious improvement on MB removal. The result indicates that as a donor of metal ions and attachment sites of Ca-MOF, the presence of CaSiO_3_ is critical to adsorption performance. There is a time-dependent adsorption property of MB on prepared Ca-MOF/ABS/TPU 3D skeleton for different MB concentrations of 50, 100 and 200 mg/L [17]. 

Figure 7a shows the plots of the equilibrium pH against the initial pH of the Ca-MOF/ABS/TPU 3D skeleton containing solution. It is clear that the equilibrium pH is kept at about 9 in the solution with the initial pH range of 3–10. The dissolution of partial Ca-MOF occurs at the initial pH ≤ 2 in the suspension due to the alkalinity of the Ca-MOF/ABS/TPU 3D skeleton. Then the equilibrium pH is gradually consistent with the initial pH and the value of pHpzc for the Ca-MOF/ABS/TPU 3D skeleton is determined as 9.3.

Figure 7b shows the pH dependence of MB sorption on the Ca-MOF/ABS/TPU 3D skeleton. In Figure 7b, the Ca-MOF/ABS/TPU 3D skeleton maintains a relatively high MB adsorption capability in either acidic or slightly alkaline conditions. However, a slight decrease in the adsorption ratio at pH ≥ 9 is observed. It is guessed that high concentration of OH^–^ in the solution may react with MB^+^ and effect the combination of Ca-MOF/ABS/TPU 3D skeleton and MB molecules. Figure 7b also shows that the initial pH has little effect on the MB adsorption for the Ca-MOF/ABS/TPU 3D skeleton. So, the adsorption experiments were performed without adjusting the initial pH. 

### 3.3. Adsorption Thermodynamics

**Figure 8:**
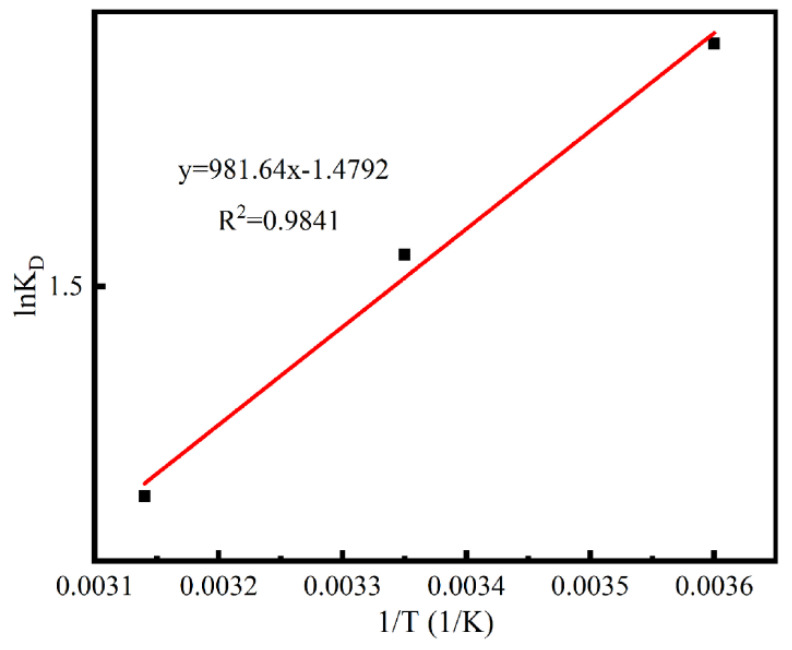
Plot of lnK_D_ versus 1/T for MB adsorption on the Ca-MOF/ABS/TPU 3D skeleton at different temperatures.

Figure 8 shows the plots of lnK_D_ against 1/T for adsorption of MB onto Ca-MOF/ABS/TPU 3D skeleton. Thermodynamic parameters such as enthalpy change (H), entropy change (S) and free energy change (G) are indices used to determine the spontaneity of a process. These three values can be calculated from the following equations:

(3) $$K_{D}=\frac{q_{e}}{C_{e}}$$

(4) $$\ln K_{D}=\frac{\Delta G}{RT}=\frac{\Delta S}{R}-\frac{\Delta H}{RT}$$

(5) $$\Delta G=-RT\ln K_{D}$$

where *K_D_* is the diffusion constant, q_e_ is the concentration of MB adsorbed at equilibrium (mg L^−1^), *C_e_* is the equilibrium concentration of MB in the solution (mg/L^−1^), *R* is the gas constant (8.314 J K^−1^ mol^−1^) and *T* is the temperature in Kelvin. The values of ∆G^0^ ∆*H*^0^ and ∆*S*^0^ were calculated and listed in Table 1.

As shown in Table 1, the negative values of of ∆H^0^ (−8.161 kJ/mol) and ∆S^0^ (−12.30 J/mol K) suggest that the adsorption process is exothermic with a decreased randomness at the solid/solution interface. Moreover, the negative values of ∆G^0^ (−4.740, −4.494 and −4.248 kJ/mol at 278.15, 298.15 and 318.15 K, respectively) confirm the spontaneous nature and feasibility of the adsorption. High temperature causes a greater positivity in the ∆G^0^ values, indicating that the adsorption process is advantageous at low temperatures. However, the value of ∆G^0^ has little change with the temperature, revealing that the temperature has a small effect on the reaction. All ∆G^0^ values are less than −10 kJ/mol, suggesting that chemistry adsorption is one of the mechanisms of MB adsorption process [34].

### 3.4. Adsorption Kinetics

**Figure 9:**
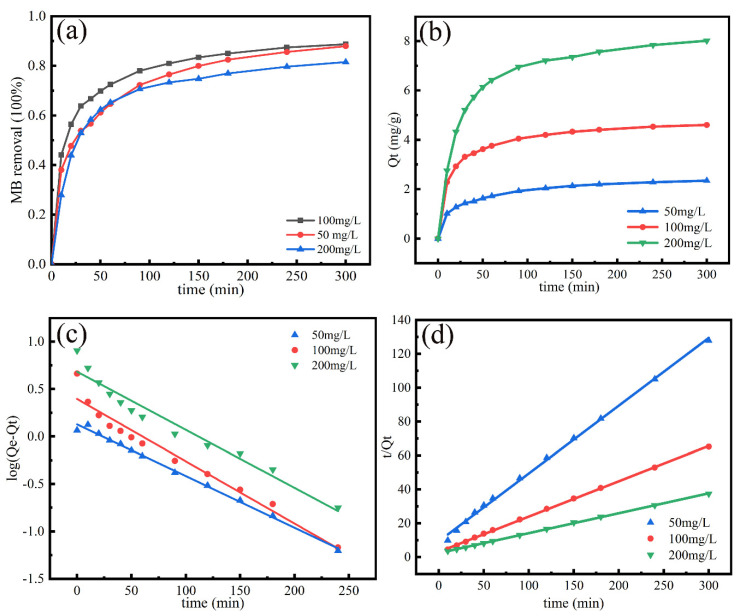
(**a**) Change of the adsorption capacity and removal efficiency with adsorption time, (**b**) *q_e_* of Ca-MOF/ABS/TPU for MB adsorption; (**c**) pseudo-first; (**d**) pseudo-second.

The adsorption kinetics experiment of MB with various initial concentrations (50, 100, 200 mg/L) on the Ca-MOF/ABS/TPU 3D skeleton were performed at 298.15 K [16]. The results are given in Figure 9. The pseudo-first-order and pseudo-second-order models are applied to analyze the experimental data for MB adsorption process (Figure 9c,d), and the calculated parameters are tabulated in Table 2. As shown in Figure 9a, the adsorption curves raise sharply when adsorption time increases from the initial one to 60 min. The MB removal ratios from aqueous solutions with various concentrations are 65%, 72% and 65% at the reaction time of 60 min. At the initial time, the presence of a large number of adsorption sites on the surface of the material leads to a rapid MB adsorption. The adsorption rate slowly increases after 60 min due to the decrement of adsorption sites by occupation of MB molecules on the surface of the material. The adsorption equilibrium is reached at 300 min and the adsorption capability at the equilibrium time is 88%, 88% and 81% respectively. Notably, 50 mg/L gives the lowest adsorption speed due to the low MB concentration. However, the MB removal efficient for 200 mg/L is found to be lower than those for 50 and 100 mg/L. The result may be attributed to the adsorption saturation of the Ca-MOF/ABS/TPU 3D skeleton at a high MB concentration. The results indicate that the proposed Ca-MOF/ABS/TPU 3D skeleton is a candidate absorbent with rapid adsorption speed, high adsorption ability for removal of dye contaminations from aqueous.

Figure 9b shows the maximum adsorption capacity of the Ca-MOF/ABS/TPU 3D skeleton in MB solution with various concentrations. It is obvious that the maximum adsorption capacity of the Ca-MOF/ABS/TPU 3D skeleton increases with the initial MB concentration. The skeleton shows a small value of q_e_ due to the low proportion of Ca-MOF (about 3.2%). The correlation coefficient (R^2^) for pseudo-second-order kinetic model is more than 0.99, whereas those for the pseudo first-order are within the ranged of 0.9667–0.9922, suggesting that pseudo-second-order is a better model to analyze the experimental data in the linearized form (Figure 9c,d) than the pseudo-first-order kinetic model. Accordingly, the adsorption rate determining step is suggested to be chemical adsorption by sharing or exchanging electrons between adsorbate and adsorbent [34].

### 3.5. Reusability

**Figure 10:**
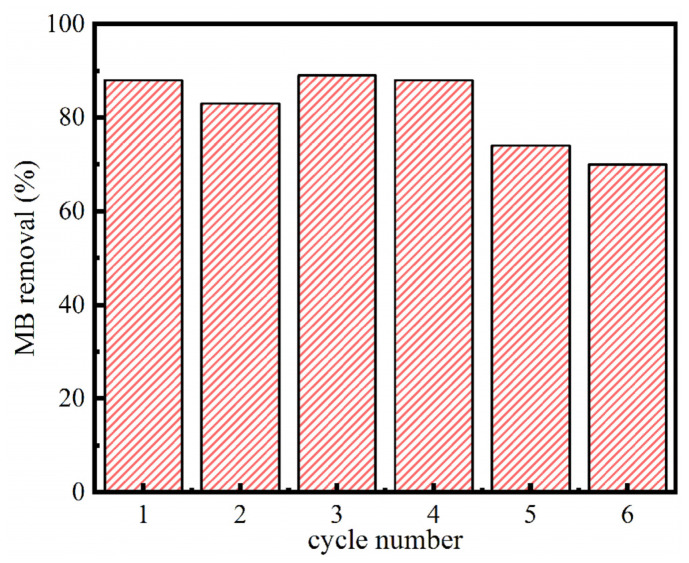
Reusability of Ca-MOF/ABS/TPU skeleton for MB removal.

The reusability of the Ca-MOF/ABS/TPU 3D skeleton was evaluated over six cycles and the results are given in Figure 10. It is observed that the removal capability gives almost no decrease by four adsorption–desorption runs. The removal ratio remains at 74% and 70% for the fifth and sixth runs, indicating that the prepared Ca-MOF/ABS/TPU 3D skeleton possessed a good reusability for MB removal. The decrement is suggested to be caused by the drop of Ca-MOF by a little extent from the skeleton surface in the process of adsorption–desorption.

## 4. Conclusions

An in-situ growth method for directly synthesizing Ca-MOF on the surface using metal in 3D printing skeleton was explored. The fabrication approach possesses advantages of shortened time and high loading rate. The MB removal ratios of the obtained Ca-MOF/ABS/TPU 3D skeleton from aqueous solution with various initial concentrations of 50, 100 and 200 mg/L are 88%, 88% and 80%, respectively, were recorded. In addition, the value remains at 70% at the sixth adsorption–desorption run, indicating that the obtained Ca-MOF/ABS/TPU 3D skeleton has high efficiency for MB removal with a good reusability. Compared with previous work [12,26], our material has advantages in treating a high concentration of printing and dyeing wastewater. Overall, a new strategy of combining MOFs and 3D printing technology to achieve powder immobilization is proposed, which may pave the way for the design of new pollutant treatment materials.

## Figures and Tables

**Table 1:** Thermodynamic parameters for the adsorption of MB on the Ca-MOF/ABS/TPU 3D skeleton.

Temperature (K)	∆G (kJ/mol)	∆H (kJ/mol)	∆S (J/mol K)
278	−4.740	−8.161	−12.30
298	−4.494		
308	−4.248		

**Table 2:** Kinetic parameters for MB adsorption on the Ca-MOF/ABS/TPU 3D skeleton.

Model	Parameter Description	Experimental Data			
		Parameters	C_0_ (mg/L)		
			50	100	200
Pseudo-first-order model: $\log(q_{e}-q_{t})=\log q_{e}-\frac{k_{1}t}{2.303}$	*q_e_*: maximum adsorption capacity, mg/g	*q_e,exp_*	2.35	4.6	8.18
		*q_e,cal_*	1.34	2.48	4.82
	*k_1_*: the rate constant of pseudo first-order adsorption, min^−1^	*k_1_*	0.01255	0.0151	0.01409
		*R^2^*	0.995	0.957	0.9492
Pseudo-second-order model: $\frac{t}{q_{t}}=\frac{1}{k_{2}q_{e}^{2}}+\frac{t}{q_{e}}$	*k_2_*: the rate constant of pseudo-second-order adsorption, (g/(mg·min))	*q_e,exp_*	2.35	4.6	8.18
		*q_e,cal_*	2.49	4.79	8.49
		*k_2_*	0.45231	0.22649	0.12686
		*R^2^*	0.9981	0.9995	0.9997
